# CRISPR-activated expression of collagen *col-120* increases lifespan and heat tolerance

**DOI:** 10.17912/micropub.biology.000730

**Published:** 2023-04-12

**Authors:** Anita Goyala, Collin Y. Ewald

**Affiliations:** 1 Laboratory of Extracellular Matrix Regeneration, Institute of Translational Medicine, Department of Health Sciences and Technology, ETH Zürich, Schwerzenbach CH-8603, Switzerland

## Abstract

Transgenic overexpression of collagen *col-120* increases the lifespan of *C. elegans* . However, whether post-developmental enhancement of collagen expression could also increase the lifespan is unknown. Recently, we described a method to induce the expression of a target gene using catalytically dead Cas9 (dCas9)-engineered *C. elegans* via ingestion of bacteria expressing a pair of promoter-specific single guide RNAs (sgRNA). Here, we cloned *col-120* promoter-specific sgRNA oligo pair into L4440-Biobrick-sgRNA and fed these bacteria to dCas9::VP64 transgenic *C. elegans* . We observed a similar percentage of lifespan extension by post-developmentally dCas9-induced expression of *col-120* , as previously reported through transgenic overexpression of *col-120* . Consistent with this result is that induction of another previously shown longevity-promoting collagen, *col-10 ,* also increased lifespan. Furthermore, we found an enhanced resilience to heat stress and increased expression of *hsp-16.2* upon dCas9-activated *col-120* expression. Together, these results provide an orthogonal method to validate longevity by enhancing *col-120* expression and point towards a potential role of collagen enhancement in thermotolerance.

**Figure 1. CRISPR-activation of collagens increases lifespan and heat stress resilience:**
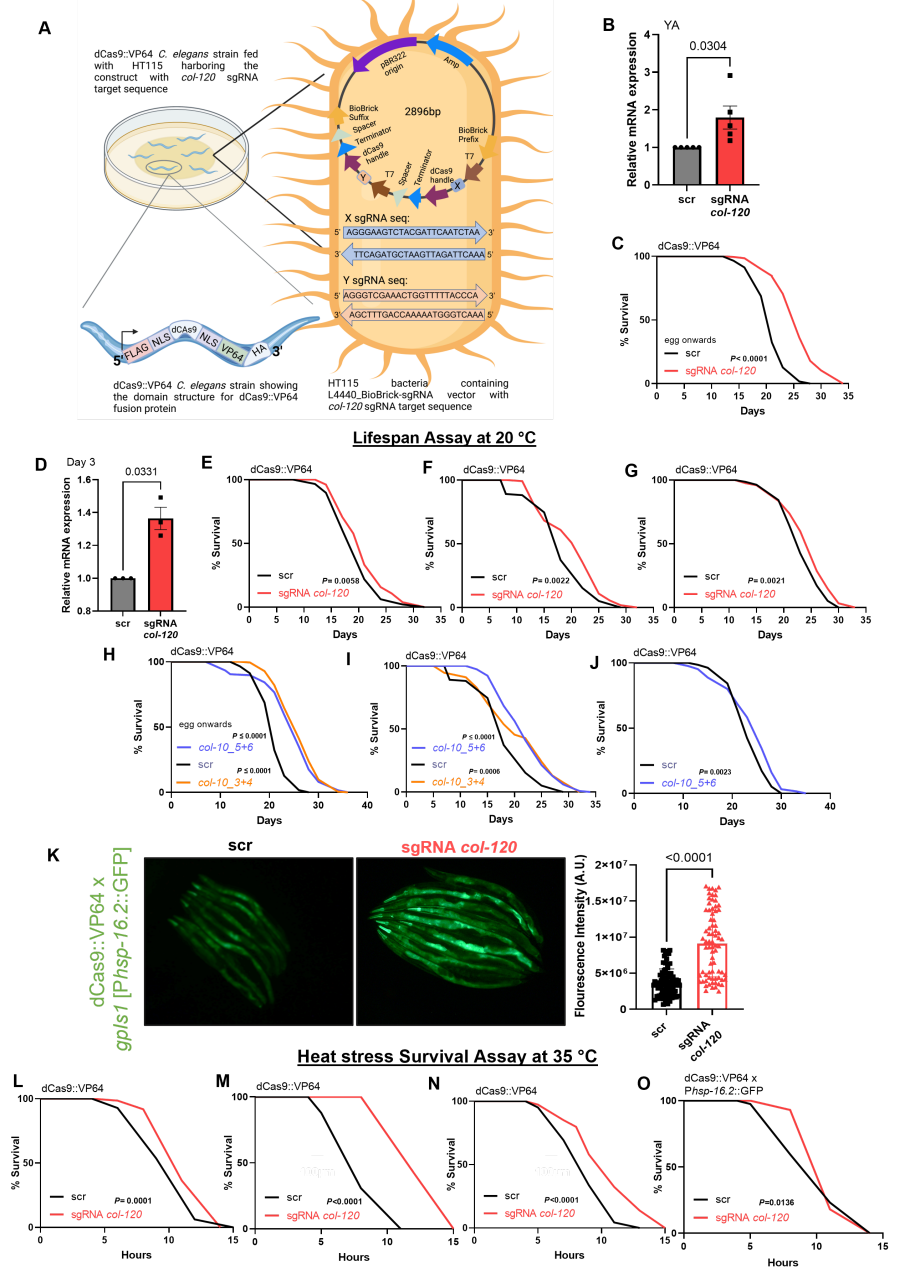
A) Schematic illustration of bacteria feeding expressing *col-120* via promoter-specific sgRNA using the L4440-Biobrick-sgRNA plasmid to dCas9::VP64 transgenic *C. elegans* . B, D) Relative fold change in mRNA expression of *col-120* gene in young adult (YA) and day three adults, respectively, when fed sgRNA *col-120* or scr to eggs (B) or L4 (D) animals. qRT-PCR experiment was performed in three or more biological replicates. Welch's two-tailed t-test was used for significance analysis. C, E-J) Survival curves showing extension in lifespan in animals with a dCas9-activated expression of *col-120* (C, E-G) and *col-10* (H-J) compared to scrambled (scr) control. C) eggs or (E-G) L4 dCas9::VP64 transgenic *C. elegans* were placed on sgRNA-expressing bacteria. Lifespans were performed at 20 °C. Experiments were performed in four independent biological trials for sgRNA *col-120* and three independent biological trials for sgRNA *col-10,* as shown individually here. The Mantel-Cox log-rank test was used for statistics. Data, details, and statistics of lifespan assays are in Extended Data. K) dCas9-activated expression of *col-120* enhanced the expression of *hsp-16.2* promoter-driven GFP after a heat shock at 33 °C for 2 hours with 6 hours of recovery at 20 °C, as shown in the representative fluorescent images. The bars represent mean + SEM and are a composite of three independent biological trials. The dots represent the quantified fluorescence intensity of P *hsp-16.2* ::GFP in arbitrary units of individual *C. elegans* . Welch’s t-test was used for significance analysis. L-O) Heat stress survival curves showed enhanced survival of animals with the dCas9-activated expression of *col-120* to high temperature (35 °C). Experiments were performed in four independent biological trials, as shown individually. The details of the heat resistance assay, summary, and statistical analysis are in Extended Data. The Mantel-Cox log-rank test was used for significance analysis.

## Description

Collagens form an extensive part of the extracellular matrix in all metazoans (Kramer, 1994) . In *C. elegans,* there are 181 collagens, categorized as cuticular and basement membrane collagens (Teuscher *et al.* 2019). Upon longevity-promoting interventions, such as reduced insulin/IGF-1 receptor signaling (rIIS), some key collagens, including *col-120* and *col-10* , showed prolonged expression during aging (Ewald *et al.* 2015). Both collagens, *col-120* and *col-10 ,* are expressed during larval development, with the highest expression at L4 but show a rapid decline in expression in young adults (Ewald *et al.* 2015; Teuscher *et al.* 2022). This progressive and age-associated decline of *col-120* mRNA and protein levels is delayed by rIIS (Teuscher *et al.* 2022). Moreover, transgenic overexpression of collagen *col-120* or *col-10* is sufficient to increase the lifespan of *C. elegans* (Ewald *et al.* 2015). However, whether post-developmental enhancement of *col-120* or *col-10* is sufficient to increase lifespan is unknown.

Several methods exist to force gene expression in *C. elegans* , such as using a heat-shock inducible promoter or a Tetracycline-controlled transcription system (Tet/Q) (Link *et al.* 1999; Bacaj and Shaham 2007; Mao *et al.* 2019). Yet, repeated heat shocks or tetracycline-analog doxycycline used for induction of the Tet/Q hybrid system can increase the lifespan of *C. elegans* (Olsen *et al* . 2006; Houtkooper *et al.* 2013; Ye *et al.* 2014; Ewald *et al* . 2016; Vertti‐Quintero *et al.* 2021; Gao *et al.* 2022). To overcome this with an orthogonal method, we employed a recently developed technique of feeding bacteria containing promoter-specific sgRNA for inducing the transcriptional expression of the endogenous target genes in a transgenic dCas9::VP64 *C. elegans* (Fischer *et al.* 2022) ( Figure 1A ). In brief, nuclease dead Cas9 (dCas9) is fused to the widely accepted transcriptional activator domain VP64, and endogenous expression of dCas9::VP64 is driven by the ubiquitous *sur-5* promoter in *C. elegans* (Fischer *et al.* 2022) ( Figure 1A ). The target gene promoter-specific sgRNAs are designed to be -50 to -400 bp upstream of the target gene's transcription start site (TTS) (Fischer *et al.* 2022). The pair of sgRNAs were cloned in L4440_BioBrick-sgRNA plasmid to induce expression in *E. coli* . Similar to RNAi, sgRNA production is induced by ITPG (Fischer *et al.* 2022). The simple cloning of specific sgRNAs and inducing gene transcription through feeding bacteria expressing the sgRNAs makes this technique easy to adapt and feasible, similar to RNAi methods.

As described in Fischer *et al.* (2022), we cloned the *col-120* (3+4 sgRNA pair) or *col-10* (3+4, 5+6 sgRNA pairs) promoter-specific sgRNA oligo sequences in two consecutive sgRNA cassettes driven by T7 promoter with BioBrick flanking region in the L4440_BioBrick-sgRNA vector. The construct was transformed into HT115 *E. coli* bacteria, and through the bacterial delivery of the sgRNA to the modified dCas9::VP64 *C. elegans* strain, the expression of *col-120* or *col-10* was induced ( Figure 1A ). The advantage of this method is that the induction of expression of the target gene can be temporarily regulated by controlling the timings of feeding to the animals.

We first placed eggs on sgRNA-expressing bacteria to dCas9-activate the expression of *col-120* ( Figure 1B ) and found a robust lifespan increase ( Figure 1C ) *.* Next, to post-developmentally enhance the expression of *col-120* , we placed L4 animals on the sgRNA-expressing bacteria, which resulted in an increase in *col-120* gene expression induction and increased lifespan compared to the control (scr = scrambled; Figure 1D -G). This validates the previously reported lifespan enhancement by transgenic COL-120 overexpression (Ewald 2015) . We also found an extension in lifespan upon feeding two different pairs of *col-10* sgRNA post-developmentally ( Figure 1H -J), further validating the longevity benefits of extracellular matrix enhancement.

One intriguing observation is that many collagen genes are highly upregulated upon short exposure to heat stress (Brunquell *et al.* 2016). Therefore, we asked whether higher collagen expression levels could be protective against heat stress. To address this, we fed *col-120* sgRNA bacteria to dCas9 transgenic animals that drive GFP expression under the heat-shock promoter *hsp-16.2* (P *hsp-16.2* ::GFP), placed them for two hours at 33 °C, and let them recover for six hours at 20 °C before quantifying GFP fluorescence. We found increased levels of *hsp-16.2* by dCas9-activated expression of *col-120* ( Figure 1K ), suggesting an enhancement of the heat shock response. Furthermore, we found that dCas9-activated expression of *col-120* enhanced survival at 35 °C ( Figure 1 L -O), suggesting that enhancing collagen expression improves heat resilience.

In summary, using dCas9-activation, we demonstrated that enhancing collagen expression promotes longevity and heat resilience. This might provide a model to study the underlying mechanisms of extracellular matrix enhancement for healthy aging.

## Methods

*Strains*

*C. elegans* strains used in this study were maintained on *E. coli* OP50 seeded nematode growth medium (NGM) plates at 20 °C. MIR249, *risIs33* [ *K03A1.5p* ::3xFLAG::SV40-NLS::dCas9::SV40-NLS::VP64::HA + *unc-119 (+)* ] and MIR276, *risIs33* [ *K03A1.5p* ::3xFLAG::SV40-NLS::dCas9::SV40-NLS::VP64::HA + *unc-119 (+)* ]; *gpIs1* [ *hsp-16.2 p* ::GFP] (Fischer *et al.* 2022).

*E. coli* strains used in this study are OP50 (streptomycin resistance) for general maintenance of *C. elegans* , HT115 (DE3) (ampicillin and tetracycline resistance) for inducing the target gene sgRNA, and DH5α (ampicillin resistance) for cloning the target gene sgRNA plasmid construct. OP50 seeded plates were prepared by spotting OP50 culture grown overnight at 37 °C from a single colony in LB broth with streptomycin antibiotic (100 µg/mL). sgRNA constructs containing HT115 bacteria primary culture were prepared by inoculating a single colony in 5 mL LB broth with ampicillin (50 µg/mL) and tetracycline (12.5 µg/mL) antibiotic and incubated at 37 °C overnight. The next day, 25 mL of LB broth with ampicillin (50 µg/mL) was added to the primary culture and let it grow at 37 °C for 3 h. Cultures were then concentrated for 15 min at 3500 rpm at room temperature. The pellet was resuspended in 5 mL LB broth with ampicillin (50 µg/mL) and Isopropyl-β-D-thiogalactopyranoside (IPTG) (3 mM), and the homogenous mix was spotted on the NGM plates. Plates were dried for one day before use.

*Cloning of col-10 and col-120 sgRNAs*

Primers for the *col-10* and *col-120* promoter-specific sgRNA target sequences were designed according to the stringent rules described in Fischer *et al.* (2022). Briefly, six best 20 nucleotide sequences flanked with NGG (sgRNA) were chosen from 50 to 400 bp region upstream of the TSS (Transcription start site) of the *col-10* or *col-120* gene, with >50 off-target scores and numbered based on their relative distance from the respective TSS, with sgRNA 1 being closest and 6 being furthest away from the TSS. Scr (scrambled) sgRNA sequences were randomly chosen 20 nucleotides with ≥50 % GC content that did not match to *C. elegans* genome upon BLAST. *col-10 (3+4, 5+6) or col-120* (3+4) promoter-specific sgRNA oligo pairs were successfully cloned into the L4440_BioBrick-sgRNA vector. Annealed oligos with appropriate overhangs of BbsI and BsaI restriction sites were ligated to the vector with BbsI and BsaI sites. Ampicillin antibiotic resistance was the selection pressure for positive ligated colonies. *E. coli* DH5α cells were used as the cloning host, and HT115 (DE3) bacteria were used for selecting and propagating the positive clone.

*Lifespan assay*

After hypochlorite treatment, eggs obtained from gravid MIR249 adults were maintained on OP50 -seeded NGM plates (except for one biological replicate where eggs were grown on scr or sgRNA *col-120 or col-10* HT115 bacteria-seeded NGM plates) until the L4 stage. 30-40 L4 animals were then transferred to scr or sgRNA *col-10* or *col-120* HT115 bacteria seeded NGM plates in replicates of five. 120 µL FUdR (final concentration of 50 µM) was top coated onto these plates. Survival of animals was assessed by gently prodding their posterior end with platinum wire to score live or dead. Unhealthy animals with vulval rupture and those crawling on the sides or under the agar were censored from the population throughout lifespan scoring. Lifespan Scoring was started at day 7 of adulthood, L4 being taken as t=0 timepoint. Survival curves were plotted using GraphPad Prism 9.2.0. Statistical analysis was performed using OASIS 2.0 software online. Lifespan summary for individual experiments is provided in Extended Data.

*Fluorescence Microscopy*

Gravid MIR276 adults were treated with sodium hypochlorite solution to obtain eggs. The eggs were grown on scr or sgRNA *col-120* HT115 bacteria seeded NGM plate until young adult. Young adults were heat-shocked at 33 °C for 2 h. After recovery from shock for 6 h at 20 °C, approx. 30 animals were mounted onto a 2 % agarose pad and anesthetized with 20 mM levamisole for imaging (Teuscher and Ewald, 2018) . Animals were stacked together and captured at 10X with one or two fields of view and then stitched together later (as shown in Figure 1K ) using ImageJ (version 1.53e). Quantifying the total fluorescence intensity by running a Python script, Green Intensity Calculator (available publicly in Github-Ewaldlab), in ImageJ (Statzer *et al.* 2021). Statistical analysis of the quantified data was done using GraphPad Prism software (GraphPad Prism 9.2.0). The experiment was performed in three biological batches, with n>80 total animals per condition.

*Heat resistance survival assay*

MIR249 (or MIR276 for Figure 1O ) gravid adults were bleached to obtain eggs and grown on OP50 -seeded NGM plates until the L4 stage. L4 animals were fed with scr and sgRNA *col-120* HT115 bacteria until adulthood day 3 at 20 °C. Day 3 animals were shifted to 35 °C to initiate heat stress. Survival of the animals was scored every 2-3 h by gently prodding their tails with a platinum wire. Survival curves were plotted using GraphPad Prism 9.2.0. Statistical analysis was performed using OASIS 2.0 software online. Survival summary for individual experiments is provided in Extended Data.

*col-120 gene expression by qRT-PCR*

Gravid MIR249 adults were treated with hypochlorite solution to obtain eggs. Eggs were either fed with *col-120* sgRNA and scr until young adult (YA) or grown on OP50 till L4 and then fed with *col-120* sgRNA and scr until day 3 of adulthood. Animals were then collected at YA and Day 3 for RNA isolation. RNA isolation was done using Trizol (Ambion #15596018) method and purified by RNA clean and concentrator kit (Zymo Research #R1017) with in-column DNase (Ambion #AM2222) digestion. 1 µg of RNA was used for first strand cDNA synthesis (SuperScript III by Invitrogen #11752-250) according to the manufacturer’s instructions. Gene expression levels were determined by quantitative real-time PCR (qRT-PCR) using the PowerUp SYBR Green MasterMix (Applied biosystems by Thermo Fischer Scientific #A25742) and QuantStudio PCR system (Applied Biosystems by Thermo Fischer Scientific) according to the manufacturer’s specifications. Relative gene expression, normalized to the geometric mean of two housekeeping genes *cdc-42* and *pmp-3* , was determined between animals treated with *col-120* sgRNA and scr. Statistical analysis was performed using GraphPad Prism 9.2.0. All the primers used are listed in Extended Data.

## Reagents

TRizol reagent (Ambion by Life Technologies) #15596018

RNA clean and concentrator kit (Zymo Research #R1017)

DNase I (RNase-Free) (Ambion #AM2222)

SuperScript III First-Strand Synthesis SuperMix for qRT-PCR (Invitrogen #11752-250)

PowerUp SYBR Green MasterMix (Applied biosystems by Thermo Fischer Scientific #A25742)

## Extended Data

Description: Extended Data File. Resource Type: Dataset. DOI: 10.22002/d1ffs-a0b42
